# Increased systemic inflammation and altered distribution of T-cell subsets in postmenopausal women

**DOI:** 10.1371/journal.pone.0235174

**Published:** 2020-06-23

**Authors:** Julie Abildgaard, Jeanette Tingstedt, Yanan Zhao, Hans Jakob Hartling, Anette Tønnes Pedersen, Birgitte Lindegaard, Susanne Dam Nielsen

**Affiliations:** 1 The Centre of Inflammation and Metabolism and the Centre for Physical Activity Research, Rigshospitalet, University of Copenhagen, København Ø, Denmark; 2 Viro-immunology Research Unit, Department of infectious diseases, Rigshospitalet, University of Copenhagen, København Ø, Denmark; 3 Virus Research and Development, Department of Virology and Special Microbial Diagnostic, Statens Serum Institut, København Ø, Denmark; 4 Department of Gynaecology, Rigshospitalet, University of Copenhagen, København Ø, Denmark; 5 Department of Pulmonary and Infectious Diseases, Nordsjællands Hospital, Hillerød, Denmark; University of Limerick, IRELAND

## Abstract

**Aim:**

To investigate markers of systemic inflammation in pre- and postmenopausal women and identify possible predictors of systemic inflammation with menopause.

**Methods:**

Cross-sectional study of 69 healthy women between 45- and 60 years. Blood samples were collected to assess leukocyte subsets and plasma cytokines. MRI and DXA scans were performed to assess body composition. Through uni- and multivariate analyses, follicle-stimulating hormone (FSH), visceral fat mass and age were evaluated as predictors of systemic inflammation in relation to menopause.

**Results:**

Postmenopausal women tended to have higher leukocyte counts (5.4 x10^9^ vs. 4.9 x10^9^ cells/l, p = 0.05) reflected in increased total lymphocytes (1.8 x10^9^ vs. 1.6 x10^9^ cells/l, p = 0.01) and monocytes (0.5 x10^9^ vs. 0.4 x10^9^ cells/l, p = 0.02), compared to premenopausal women. Increased visceral fat mass was a strong predictor of high leukocyte subsets. Postmenopausal women had higher plasma TNF-α (2.24 vs. 1.91 pg/ml, p = 0.01) and IL-6 (0.45 vs. 0.33 pg/ml, p = 0.004) compared to premenopausal women and high FSH was a significant predictor of increased plasma TNF-α, IL-1β and IL-6. Menopause was further associated with increased T-cells (1,336 vs. 1,128 cells/μl, p = 0.04) reflected in significantly higher counts of exhausted-, senescent-, and memory CD4+ T-cell subsets.

**Conclusions:**

Menopause is associated with increased systemic inflammation as well as exhausted- and senescent T-cells. We suggest, that both increased visceral fat mass and declining sex hormone levels might contribute to postmenopausal systemic inflammation and calls for further large-scale studies to confirm these findings.

## Introduction

Oophorectomy of rodents leads to increased plasma levels of pro-inflammatory cytokines including tumor necrosis factor-α (TNF-α), interleukin (IL)-6, IL-1β, IL-18, and interferon-γ (IFN-γ) and sustained increases in leukocyte counts including increased monocytes, neutrophils, and T-cells with no changes in B-lymphocyte number [1–3] all together suggesting that loss of ovarian function could be associated with chronic systemic inflammation. In accordance with this, human studies have shown that menopause is associated with increased plasma levels of IL-2 and IL-6 [4, 5]. However, the effects of menopausal status on leukocyte number and subsets are less clear and previous studies [6, 7] were performed on young premenopausal women versus 15–30 years older postmenopausal women where differences in immune function could be attributed to age.

Chronic systemic inflammation plays an important role in the etiology of metabolic disease, including insulin resistance [8], type 2 diabetes [9] and cardiovascular disease [10]—diseases known to increase in prevalence with menopause [11, 12]. Possible contributors to postmenopausal chronic inflammation are unknown, however, several mechanisms known to cause systemic inflammation could play a role.

Estrogen has been shown to prevent prolonged inflammation by directly affecting several leukocyte subsets [13, 14] suggesting that ceasing endogenous estrogen production could tip the inflammatory balance towards chronic systemic inflammation.

Furthermore, ovarian aging is closely related to chronological aging. Chronological aging is associated with chronic systemic inflammation reflected in both increased plasma IL-6 and TNF-α and progressive dysregulation of the immune response [15]. It is unclear how ovarian aging predicts systemic inflammation when controlling for chronological aging.

Lastly, as women go through menopause they experience several changes in body composition including increased visceral fat deposition [16]. In obesity, increased visceral fat mass is known to contribute substantially to chronic systemic inflammation including increased secretion of pro-inflammatory cytokines [17] and increased circulating numbers of leukocytes, T-cells, and monocytes [18]. It is unknown to what extent increased visceral fat mass contributes to postmenopausal chronic systemic inflammation.

We hypothesized that menopause was associated with increased chronic systemic inflammation reflected in increased numbers of leukocytes and plasma levels of pro-inflammatory cytokines and aimed to identify independent predictors of chronic systemic inflammation in relation to menopause. We further hypothesized that the increased chronic systemic inflammation in postmenopausal women would be reflected in changes in T-cell subsets towards cellular senescence.

## Methods

### Study design

Women were included from two different studies investigating visceral fat metabolism- (cohort A, n = 32, yet unpublished) and ectopic lipid deposition (cohort B, n = 55, [16]) in relation to menopause. A subset of the women participated in both cohort studies (n = 18). Both studies included healthy non-smoking female volunteers between 45 and 60 years of age.

Due to expenses and technical challenges cytokine analyses was only performed in cohort B (n = 55) and flow cytometry in a subset of cohort B (n = 27) (S1 Appendix).

All women went through 1) basic blood samples after an overnight fast, including white blood cell count and differential blood cell count as well as hormonal status, 2) magnetic resonance imaging (MRI) of the abdomen, and 3) DXA scan.

In women with regular menstrual bleedings, blood was sampled between day 2–8 of the menstrual cycle. Women with no menstrual bleedings during the preceding three months had blood samples taken on a random day. Plasma taken during blood sampling was used for cytokine analyses. Blood samples for flow cytometry were collected in a 9 mL EDTA tube to assess T-cell subsets in peripheral blood.

### Study participants

Women were categorized as either premenopausal (menstrual bleeding within the last 12 months) or postmenopausal (no menstrual bleeding within the last 12 months). Irregular menstrual bleedings were defined as < 10 menstrual bleedings within the last 12 months. FSH > 20 IU/l was used as a cut off to reflect some degree of decreasing ovarian function. Thus, women with an FSH > 20 IU/l was referred to as “perimenopausal”.

Exclusion criteria were: 1) chronic diseases 2) infections during the last 4 weeks, 3) smoking, 4) more than 7 alcohol units/week, 5) premature menopause (before age 40 years), 6) hysterectomy or oophorectomy prior to study inclusion, 7) BMI> 35.

### Body composition

Fat- and fat free mass were measured through DXA scanning (Lunar Prodigy Advance; GE Medical Systems Lunar, Milwaukee, WI, USA). Prodigy Software (enCORE 2004, version 8.8, GE Lunar Corp., Madison, WI, USA) was used to estimate total fat and fat-free tissue masses.

### MRI of the abdomen

MRIs were performed using a Siemens Magnetom Prisma 3 Tesla matrix magnetic resonance scanner (Erlangen, Germany) at 3 mm intervals. The method has been described previously [16]. Briefly, all adipose tissue located from the diaphragm to pelvic floor inside the peritoneum was traced manually. Software (Mango Multi-Image Analysis, Research Imaging Institute, Houston, Texas) was used to calculate the total volume of visceral fat from the T1 weighted MRI sequence. A single reader blinded to the subjects’ menopausal status performed all analyses.

### Laboratory analyses

All blood samples were analyzed at the Department of Clinical Biochemistry, Rigshospitalet, Denmark. EDTA Plasma tubes were immediately spun at 3500 g for 15 min at 4° C (except when used for flow cytometry).

White blood cell counts were analyzed on all 69 subjects, independent of flow cytometry gating procedures at the Department of Clinical Biochemistry, Rigshospitalet, Copenhagen, Denmark.

### Cytokine analyses

Plasma cytokine levels were determined in duplicates using the V-plex pro-inflammatory panel (MSD, Rockville, MD, USA). Detection ranges were: interferon-γ (IFN-γ) (0.05–0.62 pg/ml), tumor necrosis factor-α (TNF-α) (0.01–0.13 pg/ml), interleukin-1β (IL-1β) (0.01–0.27pg/ml), IL-2 (0.01–0.29 pg/ml), IL-6 (0.01–0.11 pg/ml), IL-10 (0.01–0.15 pg/ml), IL-17 (0.74–3,653 pg/ml), IL-18 (275–1,033pg/ml). Inter-assay coefficients of variations were below 10%.

### Flow cytometry

In brief, 100 μL of EDTA blood was incubated with fluorescent dye-conjugated monoclonal antibodies (BD Bioscience, Franklin Lakes, NJ, USA) at room temperature for 20 minutes according to the manufacturer’s protocol (BD Bioscience, Franklin Lakes, NJ, USA). Erythrocytes were then lysed using 2 ml of lysing solution (BD Bioscience, Franklin Lakes, NJ, USA) and incubated for 10 minutes. Lastly, cells were washed, centrifuged at 300g for 5 minutes and re-suspended in PBS. Monoclonal antibodies used to determine T-cell subsets were: CD3 (peridinin chlorophyll proteins-cyanine (PerCP), clone SK7), CD4 (Brilliant Violet 510 (BV510), clone SK3), CD8 (Flourescein isothiocyanate (FITC), clone HIT28a), CD28 (phycoerythrin cyanine 7 (PE-Cy7), clone CD28.2), CD95 (BV421, clone DX2), CD57 (Allophycocyanin (APC), clone NK-1), CD38 (PE-Cy7, clone HIT2), CD45RA (PE, clone HI100), CD25 (PE-Cy7, clone M-A251), CD127 (BB515, clone HIL-7R-M21), CCR4 (PE, clone 1G1), CCR6 (APC, clone 11A9), CCR7 (BV421, clone 150503), HLA-DR (APC, clone G46-6), PD-1 (PE, clone MIH-4) and FVS780 (APC-Cy7). Six-color acquisition was performed using FACS Canto (BD Bioscience, Franklin Lakes, NJ, USA) and data processing was done using FlowJo v10.4 (FlowJo, LLC, Ashland, OR, USA).

### Gating strategy

Gating strategies are shown in S2 Appendix. FMO controls were used to ensure proper gating. Absolute lymphocyte counts were determined by multiplying the proportion of a specific subset in the lymphocyte gate by the total lymphocyte count.

### Power calculations

Power calculations were based on previous studies showing chronic systemic inflammation with increased visceral fat deposition. Based on previous studies [16, 19] we expected around a 100% increase in visceral fat mass in postmenopausal women compared to premenopausal women, which should be adequate to detect a 15% increase in leukocytes (standard deviation, SD = 0.2) [18] and around a 20% increase in inflammatory cytokines (SD = 0.25) [17, 20]. α was set to 0.05 and β = 0.2. Sample sizes were calculated to at least n = 56 for leukocyte analyses and n = 50 for cytokine analyses.

### Statistical analyses

All data are presented as medians (inter quartile range, IQR) unless otherwise stated. Comparisons between pre- and postmenopausal women were evaluated using an unpaired t-test. To achieve normal distribution variables were log-transformed if necessary. If normal distribution could not be achieved through log-transformation comparisons were performed using a Mann-Whitney U test. Regression models were checked for assumptions of the linear model including normal distribution of the residuals, homogeneity of variance, linearity and independent observations. Standardized beta (β_S_) was used to assess the impact of the predictors in the multivariate analyses. Outliers outside 3 inter quartile ranges from the median were excluded from the statistical analyses (IL-1β (n = 1), TNF-α (n = 1), plasma IL-6 (n = 1), IFN-γ (n = 2), CD4^+^ & CD8^+^ T-cells (n = 1)) as these were assumed to reflect acute inflammation. Cytokines with a left truncated distribution due to values below detection range (IL-1β, IL-2) were analyzed using a tobit regression, which is designed to estimate linear relationships between variables when there is left censoring in the dependent variable.

In addition to the predefined statistical analysis plan, exploratory analyses on the correlation between inflammatory cytokines and number of exhausted- and senescent T-cells was performed using a Spearman correlation.

Statistical analyses were performed using IBM SPSS statistics version 22. Tobit regressions were performed using Stata MP 15. P-values < 0.05 were considered statistically significant.

Informed consent was obtained in writing and verbally from all participants. The study was approved by the ethical committee of the Capitol Region, Denmark (H-3-2014-096) and performed according to the declaration of Helsinki.

## Results

### Subject characteristics

Postmenopausal women (N = 31) had a median age of 55 years (range: 50–60) and had been postmenopausal for 5 years (IQR: 3–7). Premenopausal women (N = 38) were younger than postmenopausal women and had a median age of 49 years (range: 45–55) (p = 0.001). Postmenopausal women had significantly lower serum levels of estradiol (57 pmol/l (36–69) vs. 140 pmol/l (53–360), p = 0.0003) and higher FSH (67.8 IU/l (57.5–88.7) vs. 13.3 IU/l (6.6–53.3), p<0.0001) compared to premenopausal women. Within the premenopausal group, eleven of the women were perimenopausal with a serum FSH > 20IU/l. Six of the women also had irregular menstrual bleedings.

Postmenopausal women had a larger total fat mass (24.3 kg (18.7–29.1) vs. 20.5 kg (15.9–24.6), p = 0.04) and visceral fat mass (0.70 L (0.34–1.24) vs. 0.33 L (0.21–0.46), p = 0.001) (Table 1).

**Table 1:** Subject characteristics of pre- and postmenopausal women.

	Premenopausal	Postmenopausal	p-value
N	38	31	
Age, years (range)	49 (45–55)	55 (50–60)	0.001
***Female sex hormones***			
Estradiol, pmol/l	140 (53–360)	57 (36–69)	0.0003
FSH, IU/l	13.3 (6.6–53.3)	67.8 (57.5–88.7)	<0.0001
***Body composition***			
Height, cm	170 (165–174)	167 (163–172)	0.38
Weight, kg	67.4 (60.2–70.6)	70 (61.2–78.7)	0.21
BMI	23.2 (21.4–24.7)	25.3 (21.9–27.5)	0.06
Fat mass, kg	20.5 (15.9–24.6)	24.3 (18.7–29.1)	0.04
Visceral fat mass, l	0.33 (0.21–0.46)	0.70 (0.34–1.24)	0.001
***White blood cell counts***			
Leukocytes, x10^9^cells/l	4.9 (4.2–6.0)	5.4 (4.6–6.3)	0.05
Total lymphocytes, x10^9^cells/l	1.6 (1.4–1.9)	1.8 (1.5–2.2)	0.01
Monocytes, x10^9^cells/l	0.4 (0.3–0.5)	0.5 (0.4–0.5)	0.02
Neutrophils, x10^9^cells/l	2.6 (1.8–3.2)	2.8 (2.4–3.5)	0.08
Neutrophil/lymphocyte ratio, AU	1.7 (1.0–2.7)	1.4 (1.2–2.2)	0.92

Data presented as median (interquartile range) unless otherwise stated. N, number of subjects. FSH, follicle stimulating hormone. AU, arbitrary units. Variables in pre-and postmenopausal women were compared using an unpaired t-test. If normal distribution could not be achieved through logarithmic transformation comparisons were performed using a Mann-Whitney U test.

Representative subsets of the full study group were used for cytokine analyses (n = 55: 30 premenopausal women (median age: 48 years) and 25 postmenopausal women (median age: 55 years)) and flow cytometric analyses (n = 27: 15 premenopausal women (median age: 47 years) and 12 postmenopausal women (median age: 55 years)) (See S1 Appendix for subject characteristics of subgroups).

### White blood cell counts

Postmenopausal women tended to have higher leukocyte counts (5.4 x10^9^ cells/l (4.6–6.3) vs. 4.9 x10^9^ cells/l (4.2–6.0), p = 0.05) reflected in a significantly higher total lymphocyte count (1.8 x10^9^ cells/l (1.5–2.2) vs. 1.6 x10^9^ cells/l (1.4–1.9), p = 0.01) and monocyte count (0.5 x10^9^ cells/l (0.4–0.5) vs. 0.4 x10^9^ cells/l (0.3–0.5), p = 0.02), compared to premenopausal women. There were no significant differences in neutrophil counts between post- and premenopausal women (2.8 x10^9^ cells/l (2.4–3.5) vs. 2.6 x10^9^ cells/l (1.8–3.2), p = 0.08) and neutrophil:lymphocyte ratio was comparable across menopausal status (1.4 (1.2–2.2) vs. 1.7 (1.0–2.7), p = 0.92) (Table 1).

Univariate analyses showed no associations between FSH and leukocytes. Visceral fat mass was a strong predictor of both leukocyte- (β: 0.16, 95% CI: 0.09–0.22, p<0.0001), total lymphocyte- (β: 0.10, 95% CI: 0.02–0.17, p = 0.01), monocyte- (β: 0.13, 95% CI: 0.05–0.22, p = 0.002), and neutrophil counts (β: 0.20, 95% CI: 0.10–0.30, p = 0.0002) as well as neutrophil: lymphocyte ratio (β: 0.15, 95% CI: 0.02–0.27, p = 0.02). Age was a significant predictor of total lymphocytes (β: 0.84, 95% CI: 0.12–1.56, p = 0.02) and monocytes (β: 1.20, 95% CI: 0.41–1.99, p = 0.003) (Table 2). Multivariate analyses with FSH, visceral fat mass, and age as predictors of leukocyte subsets, showed that visceral fat mass remained a strong predictor of leukocyte-, total lymphocyte-, monocyte-, and neutrophil counts and neutrophil: lymphocyte ratio (Table 3).

**Table 2:** Univariate regression analyses of predictors of white blood cell counts.

Dependent variables:	Univariate analyses					
	Predictors:					
	FSH		Visceral fat mass		Age	
	β_U_ (95% CI)	p	β_U_ (95% CI)	P	β_U_ (95% CI)	p
**Leukocytes**	-0.01 (-0.06–0.05)	0.82	0.16 (0.09–0.22)	<0.0001	0.45 (-0.25–1.15)	0.20
**Total lymphocytes**	0.04 (-0.02–0.09)	0.18	0.10 (0.02–0.17)	0.01	0.84 (0.12–1.56)	0.02
**Monocytes**	0.03 (-0.03–0.09)	0.32	0.13 (0.05–0.22)	0.002	1.20 (0.41–1.99)	0.003
**Neutrophils**	-0.01 (-0.09–0.07)	0.84	0.20 (0.10–0.30)	0.0002	0.71 (-0.33–1.75)	0.18
**Neutrophil: lymphocyte ratio**	-0.03 (-0.11–0.06)	0.51	0.15 (0.02–0.27)	0.02	-0.39 (-1.51–0.74)	0.50

Univariate regression analyses of predictors of white blood cell counts in pre- (n = 38) and postmenopausal women (n = 31). FSH, follicle stimulating hormone. β_U_, Unstandardized β-values. CI, confidence interval.

**Table 3:** Multivariate regression analyses of predictors of white blood cell counts.

Dependent variables:	Multivariate analyses								
	Predictors:								
	FSH			Visceral fat mass			Age		
	β_S_	β_U_ (95% CI)	p	β_S_	β_U_ (95% CI)	p	β_S_	β_U_ (95% CI)	p
**Leukocytes**	-0.29	-0.06 (-0.12–0.01)	0.06	0.54	0.16 (0.09–0.23)	<0.0001	0.15	0.44 (-0.45–1.33)	0.33
**Total lymphocytes**	-0.06	-0.01 (-0.08–0.06)	0.71	0.23	0.07 (-0.01–0.15)	0.08	0.23	0.71 (-0.36–1.77)	0.19
**Monocytes**	-0.26	-0.07 (-0.15–0.01)	0.10	0.27	0.10 (0.01–0.19)	0.02	0.43	1.49 (0.35–2.62)	0.01
**Neutrophils**	-0.29	-0.09 (-0.19–0.01)	0.07	0.44	0.20 (0.09–0.30)	0.0005	0.20	0.88 (-0.54–2.30)	0.22
**Neutrophil: lymphocyte ratio**	-0.12	-0.51 (-2.17–1.14)	0.53	0.38	0.18 (0.05–0.32)	0.008	-0.14	-0.56 (-2.24–1.11)	0.50

Multivariate regression analyses of predictors of white blood cell counts in pre- (n = 38) and postmenopausal women (n = 31).

FSH, follicle stimulating hormone. β_S_, Standardized β-values. β_U_, Unstandardized β-values. CI, confidence interval.

### Cytokines

Plasma cytokines were analyzed on 55 study participants. Eleven women (7 pre- and 4 postmenopausal) had IL-1β- and nine (6 pre- and 3 postmenopausal) had IL-2 below the lower limit of detection.

Postmenopausal women had significantly higher plasma IL-6 (0.45 (0.36–0.54) vs. 0.33 pg/ml (0.24–0.41), p = 0.004) and TNF-α (2.24 pg/ml (1.92–2.37) vs. 1.91 pg/ml (1.51–2.36), p = 0.01) compared to premenopausal women. There were no significant differences in plasma IFN-γ, IL-1β, IL-2, IL-10, IL-17, and IL-18 between pre- and postmenopausal women (Table 4).

**Table 4:** Plasma cytokine levels in pre- and postmenopausal women.

	Premenopausal	Postmenopausal	p-value
**IFN-γ (pg/ml)**	2.20 (1.50–3.38)	3.36 (2.20–4.85)	0.11
**IL-1β (pg/ml)**	0.025 (0.010–0.036)	0.034 (0.020–0.059)	0.16
**IL-2 (pg/ml)**	0.10 (0.07–0.14)	0.11 (0.09–0.14)	0.26
**IL-6 (pg/ml)**	0.33 (0.24–0.41)	0.45 (0.36–0.54)	0.004
**IL-10 (pg/ml)**	0.16 (0.12–0.23)	0.18 (0.15–0.20)	0.70
**IL-17 (pg/ml)**	0.82 (0.45–0.96)	0.70 (0.50–1.13)	0.54
**IL-18 (ng/ml)**	0.41 (0.35–0.52)	0.41 (0.37–0.46)	0.60
**TNF-α (pg/ml)**	1.91 (1.51–2.36)	2.24 (1.92–2.37)	0.01

Plasma cytokine levels in pre- (n = 30) and postmenopausal women (n = 25). Values are means (Interquartile range). Plasma cytokine levels in pre-and postmenopausal women were compared using an unpaired t-test. If normal distribution could not be achieved through logarithmic transformation comparisons were performed using a Mann-Whitney U test.

Univariate analyses showed, that FSH was a significant predictor of plasma IL-1β (β: 8.81, 95% CI: 1.35–16.26, p = 0.02), IL-6 (β: 0.17, 95% CI: 0.06–1.98, p = 0.003), and TNF-α (β: 1.45, 95% CI: 0.46–2.44, p = 0.005). Visceral fat mass was not significantly associated with any plasma cytokines. Age was a significant predictor of IL-6 (β: 1.90, 95% CI: 0.44–3.35, p = 0.01) and TNF-α (β: 1.13, 95% CI: 0.13–2.13, p = 0.03) (Table 5). In multivariate analyses with FSH, visceral fat mass and age as predictors of plasma cytokines, FSH remained the strongest predictor of IL-1β, IL-6, and TNF-α, although statistical significance was abolished (Table 6).

**Table 5:** Univariate regression analyses of predictors of plasma cytokine levels.

Dependent variables:	Univariate analyses					
	Predictors:					
	FSH		Visceral fat mass		Age	
	β (95% CI)	P	β (95% CI)	P	β (95% CI)	p
**IFN-γ**	1.83 (-1.34–4.99)	0.25	0.14 (-0.10–0.37)	0.24	2.24 (-0.76–5.23)	0.14
**IL-1β**	8.81 (1.35–16.26)	0.02	0.35 (-0.23–0.92)	0.23	3.02 (-0.20–6.23)	0.07
**IL-2**	8.33 (-2.67–19.34)	0.14	-0.45 (-1.30–0.40)	0.29	4.37 (-0.17–8.91)	0.06
**IL-6**	0.17 (0.06–0.28)	0.003	0.11 (-0.01–0.22)	0.08	1.90 (0.44–3.35)	0.01
**IL-10**	1.24 (-1.04–3.52)	0.28	0.02 (-0.15–0.19)	0.80	1.04 (-1.17–3.25)	0.35
**IL-17**	-0.06 (-2.11–1.98)	0.96	0.12 (-0.03–0.27)	0.12	0.55 (-1.45–2.54)	0.58
**IL-18**	0.36 (-0.53–1.25)	0.42	0.04 (-0.02–0.11)	0.20	0.13 (-0.74–1.00)	0.77
**TNF-α**	1.45 (0.46–2.44)	0.005	0.04 (-0.04–0.12)	0.30	1.13 (0.13–2.13)	0.03

Univariate regression analyses of predictors of plasma cytokine levels in pre- (n = 30) and postmenopausal women (n = 25).

FSH, follicle stimulating hormone. β_U_, Unstandardized β-values. CI, confidence interval.

**Table 6:** Multivariate regression analyses of predictors of plasma cytokine levels.

Dependent variables:	Multivariate analyses								
	Predictors:								
	FSH			Visceral fat mass			Age		
	β_S_	β_U_ (95% CI)	p	β_S_	β_U_ (95% CI)	p	β_S_	β_U_ (95% CI)	p
**IFN-γ**	0.03	0.29 (-4.70–5.28)	0.91	0.12	0.10 (-0.15–0.35)	0.42	0.16	1.67 (-3.23–6.57)	0.50
**IL-1β**	0.31	3.01 (-1.09–7.11)	0.14	0.09	0.10 (-0.22–0.42)	0.55	-0.01	-0.05 (-4.17–4.07)	0.98
**IL-2**	-0.02	-0.25 (-6.42–5.92)	0.94	-0.19	-0.30 (-0.78–0.18)	0.21	0.34	4.81 (-1.36–10.98)	0.12
**IL-6**	0.34	0.14 (-0.02–0.31)	0.09	0.21	0.14 (-0.05–0.30)	0.14	0.03	0.14 (-2.09–2.36)	0.90
**IL-10**	0.13	1.01 (-2.58–4.61)	0.57	0.02	0.01 (-0.26–0.29)	0.92	0.03	0.26 (-3.34–3.85)	0.89
**IL-17**	-0.15	-1.09 (-4.26–2.09)	0.50	0.19	0.15 (-0.09–0.40)	0.22	0.13	0.93 (-2.25–4.11)	0.56
**IL-18**	0.20	0.65 (-0.69–1.99)	0.34	0.19	0.07 (-0.04–0.18)	0.20	-0.18	-0.56 (-1.93–0.81)	0.42
**TNF-α**	0.36	1.37 (-0.14–2.89)	0.07	0.11	0.05 (-0.08–0.17)	0.45	0.00	-0.02 (-1.56–1.53)	0.98

Multivariate regression analyses of predictors of plasma cytokine levels in pre- (n = 30) and postmenopausal women (n = 25).

FSH, follicle stimulating hormone. β_U_, Unstandardized β-values. CI, confidence interval.

Excluded cytokine outliers were measured in two premenopausal women both with an increased FSH between 60–75 IU/L (thus belonging to the “perimenopausal” category). Whereas menopausal mean differences in cytokine levels between pre- and postmenopausal women were affected by the exclusion of the outliers, the univariate- and multivariate analyses did not change considerably with the exclusion.

### T-cell subsets in pre- and postmenopausal women

**Fig 1:**
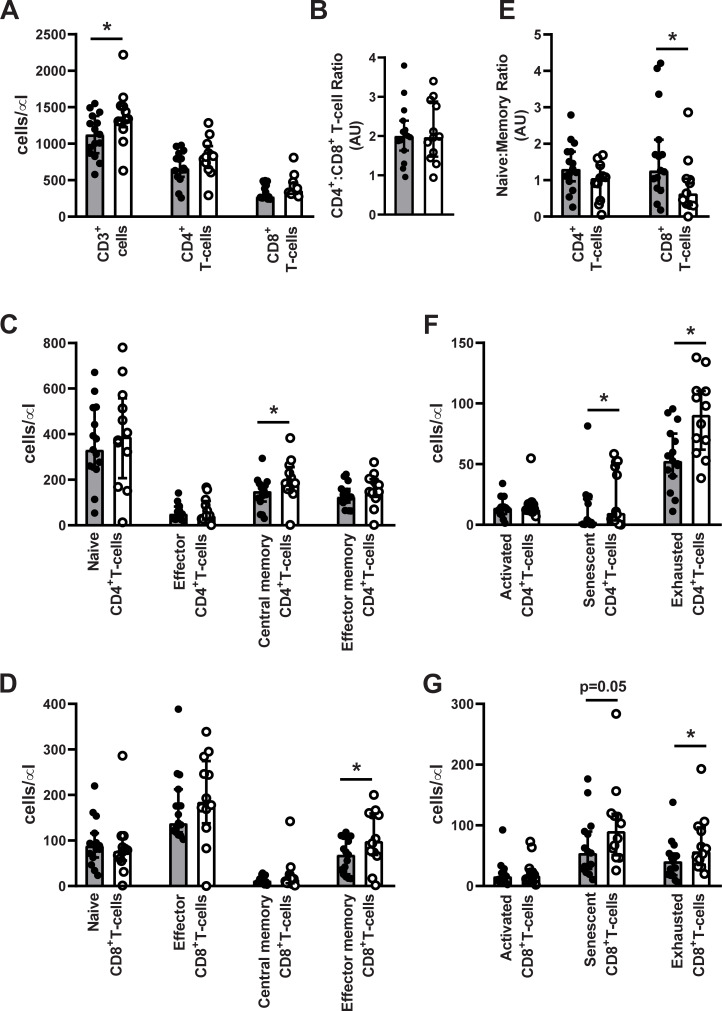
T-cell subsets in pre-and postmenopausal women. Premenopausal (n = 15, grey bars and black dots) and postmenopausal (n = 12, white bars and white dots) women. (A) CD3+ lymphocytes, CD4+ T-cells, and CD8+ T-cells. (B) CD4+:CD8+ T-cell ratio. (C) Naïve- (CD45RA+CCR7+), effector- (CD45RA+CCR7-) and central memory- (CM, CD45RA-CCR7+) and effector memory (EM, CD45RA-CCR7-) CD4+ T-cells. (D) Naïve- (CD45RA+CCR7+), effector- (CD45RA+CCR7-) and central memory- (CM, CD45RA-CCR7+) and effector memory (EM, CD45RA-CCR7-) CD8+ T-cells. (E) Naïve to memory CD4+ and CD8+ T-cell ratio. (F) Activated- (CD38+HLA-DR+), senescent- (CD28-CD57+), and exhausted (PD-1+) CD4+ T-cells. (G) Activated- (CD38+HLA-DR+), senescent- (CD28-CD57+), and exhausted (PD-1+) CD8+ T-cells. Data are presented as median (IQR). *Significantly different from premenopausal women, p<0.05.) T-cell counts in pre-and postmenopausal women were compared using an unpaired t-test. If normal distribution could not be achieved through logarithmic transformation comparisons were performed using a Mann-Whitney U test.).

T-cell subsets were analyzed on 27 study participants. Postmenopausal women had a significantly higher number of circulating CD3+ lymphocytes (1,336 cells/μl (1,253–1,515) vs. 1,128 cells/μl (820–1,380), p = 0.04) compared to premenopausal women (Fig 1A). However, post- and premenopausal women had comparable total CD4+ and CD8+ T-cell counts (Fig 1A) and CD4+:CD8+ T-cell ratio did not differ between pre- and postmenopausal women (2.01 (1.17–3.80) vs. 1.97 (0.94–3.40)) (Fig 1B).

### Production and maturation of T-cells in post- and premenopausal women

Pre- and postmenopausal women showed no differences in absolute naïve CD4+ and CD8+ T-cell counts and effector CD4+ and CD8+ T-cell counts. Postmenopausal women had higher absolute numbers of central memory CD4+ T-cells (185 cells/μl (157–246) vs. 150 cells/μl (105–183), p = 0.04) and effector memory CD8+ T-cells (99 cells/μl (70–159) vs. 69 cells/μl (26–109), p = 0.04) compared to premenopausal women (Fig 1C and 1D). Postmenopausal women showed borderline significant differences in naïve: memory CD4+ T-cell ratio (1.06 (0.44–1.41) vs. 1.31 (0.97–1.78), p = 0.07) and had a significantly lower naïve: memory CD8+ T-cell ratio (0.64 (0.32–1.04) vs. 1.27 (0.78–2.11), p = 0.03) compared to premenopausal women (Fig 1E).

### Activated and exhausted T-cells in post- and premenopausal women

Post- and premenopausal women had comparable total numbers of activated CD4+ and CD8+ T-cells. Postmenopausal women had higher numbers of senescent CD4+ T-cells (10 cells/μl (4–50) vs. 3 cells/μl (1–21), p = 0.001) and tended to have higher numbers of senescent CD8+ T-cells (91 cells/μl (54–118) vs. 54 cells/μl (28–90), p = 0.05) compared to premenopausal women. The absolute number of exhausted CD4+ (91 cells/μl (65–110) vs. 53 cells/μl (40–75), p = 0.005) and CD8+ T-cells (57 cells/μl (38–94) vs. 41 cells/μl (18–54), p = 0.04) was significantly higher in postmenopausal women compared to premenopausal women (Fig 1F and 1G). As an exploratory analysis we tested if increased plasma cytokine levels predicted number of senescent- and exhausted T-cells. Neither plasma IL-6- nor TNF-α levels predicted number of exhausted or senescent CD4+ or CD8+ T-cells (Data not shown).

Due to limited power in the flow cytometry analyses we were unable to perform multiple regression analyses to assess important predictors of T-cell exhaustion and -senescence with adequate precision.

## Discussion

The main findings of this study were that postmenopausal women showed increased total lymphocyte- and monocyte- counts, as well as increased plasma IL-6 and TNF-α. Furthermore, menopause was associated with an increased T-cell count and significantly higher counts of exhausted, senescent- and memory T-cell subsets all together indicating that menopause might be associated with increased chronic systemic inflammation.

The increased total lymphocyte- and monocyte counts in postmenopausal women are in accordance with animal studies where oophorectomy has been shown to lead to T-cell expansion and adipose tissue macrophage infiltration [1, 2]. In our study, visceral fat mass was a very strong predictor of all leukocyte subsets. This could indicate that the increased leukocyte counts seen after menopause are not directly related to the decreasing levels of female sex hormones but rather to the changes in body composition towards increased visceral fat deposition. In accordance with this, previous studies agree that abdominal obesity is related to increased circulating total lymphocyte- and monocyte counts contributing to systemic inflammation [21, 22]. It is unknown if menopause further leads to increased immune cell infiltration of the adipose tissue as is seen in studies in rodents following oophorectomy [23].

In this study, postmenopausal women showed increased plasma levels of IL-6 and TNF-α compared to premenopausal women and FSH was a significant predictor of IL-1β, IL-6 and TNF-α. The 36% increase in plasma IL-6 in postmenopausal women was robust and is in accordance with previous studies [4, 5]. At the same time, plasma IL-6 has been shown to be significantly positively associated with chronological aging [24] and, to our knowledge, no previous study has aimed to investigate the association between ovarian aging and plasma IL-6 after controlling for age. Controlling for age in this study, reduced the association between FSH and IL-6 and significance was abolished suggesting that at least some of the effect of increased pro-inflammatory cytokines following menopause could be mediated by age.

Visceral fat mass was not a strong predictor of plasma cytokine levels in this study population. In accordance with this, TNF-α produced in the adipose tissue is hypothesized to work in a more paracrine fashion and the visceral adipose tissue contribution to systemic TNF-α levels is believed to be limited [25]. High plasma IL-6 has previously been associated with increased visceral fat mass in obese populations [20]. However, it is possible, that the relatively low variability in both visceral fat mass and plasma IL-6 in this healthy cohort of women diminished this association.

Postmenopausal women had increased numbers of memory-, senescent- and exhausted T-cells compared to premenopausal women, further suggesting that menopause is associated with increased immune senescence. In accordance with this, studies in both rodents [3] and rhesus monkeys [26] showed premature T-cell senescence following oophorectomy. Furthermore, increased levels of TNF-α and IL-6 have been shown to lead to premature senescence of T-cells and blocking of TNF-α signaling prevents T-cell senescence [27] indicating that perhaps the increased plasma levels of cytokines following menopause could contribute to the increased number of senescent T-cells observed in postmenopausal women. However, in this group of women, plasma TNF-α and IL-6 levels did not predict senescent or exhausted T-cell numbers, which could be a matter of limited power in the analyses or the fact that several other molecular- and cellular mechanisms are known to contribute to T-cell senescence and -exhaustion [28].

Increased transition from memory- to exhausted- and senescent T-cells leads to a loss of function necessary for adequate immune protection and replicative capacity [29]. Thus, T-cell exhaustion and–senescence may prevent optimal control and clearance of infections [30]. In accordance with this, postmenopausal women show impaired response to vaccines and tend to have exacerbated courses of specific infections [31]. However, the clinical role for accelerated immune senescence in relation to the menopausal transition is not well described.

A high neutrophil: lymphocyte ratio and a low CD4+:CD8+ T-cell ratio have both been suggested as markers of systemic inflammation and have been associated with an increased mortality rate in comorbid aging humans [32–34]. In this study, both neutrophil: lymphocyte ratio and CD4+:CD8+ T-cell ratios were comparable across menopausal status. The clinical impact of these ratios is less well described in younger otherwise healthy populations, however, our findings could indicate that the degree of systemic inflammation, at least at this relatively early stage of menopause, after all is relatively modest.

Our study has some limitations. We estimated FSH, visceral fat mass and aging as predictors of chronic systemic inflammation in pre- and postmenopausal women. However, we cannot exclude that parameters not considered in this study may confound these estimates. For example, menopause is associated with increased insulin resistance [16, 19]. As insulin resistance is closely reciprocally related to systemic low-grade inflammation, we speculate that insulin resistance might also contribute to low-grade inflammation following menopause.

Early follicular phase FSH has been shown to be a good predictor of reproductive aging [35]. Thus, due to less variability in relation to the menstrual cycle and higher sensitivity of locally available FSH-laboratory analyses, compared to estradiol, we used early follicular phase FSH as a measure of circulating female sex hormones. FSH was measured on a random day in women with no menstrual bleedings in three months preceding study inclusion. Thus, we cannot exclude that the impact of FSH as a predictor of systemic inflammation with menopause has been underestimated due to some degree of randomness in the sampling of FSH in this subgroup of women. Eleven of the premenopausal women had increased FSH reflecting a perimenopausal state with decreasing ovarian function. Thus, the inclusion of these women in the premenopausal group might further underestimate menopausal differences in the studied variables.

The recruitment was based on convenience sampling with the associated risk of selection bias. We did not adjust for multiple testing which imposes the risk of a type 1 error. However, due to the explorative nature of this study and based on adherence to a predefined statistical analysis plan, we chose to report data without adjusting for multiple testing and let the reader interpret the analyses as exploratory. However, the reader should keep the risk of type 1 error in mind.

We conclude, that menopause is associated with increased total lymphocyte- and monocyte counts, including higher counts of exhausted, senescent- and memory T-cell subsets, as well as increased plasma IL-6 and TNF-α. We suggest, that both increased visceral fat mass and declining sex hormone levels might contribute to postmenopausal systemic inflammation and calls for further large-scale studies to confirm these findings.

## Supporting information

S1 Appendix Study groups and subject characteristics. (DOCX)

S2 Appendix Gating strategies. (DOCX)
