# Supplementary material for: Increased systemic inflammation and altered distribution of T-cell subsets in postmenopausal women
Source: PLoS One. 2020 Jun 23;15(6):e0235174. doi: 10.1371/journal.pone.0235174 (PMC7310708; doi:10.1371/journal.pone.0235174)
Supplement: S1 Appendix — (DOCX) [file pone.0235174.s001.docx]

**S1 APPENDIX: STUDY GROUPS AND SUBJECT CHARACTERISTICS**

**
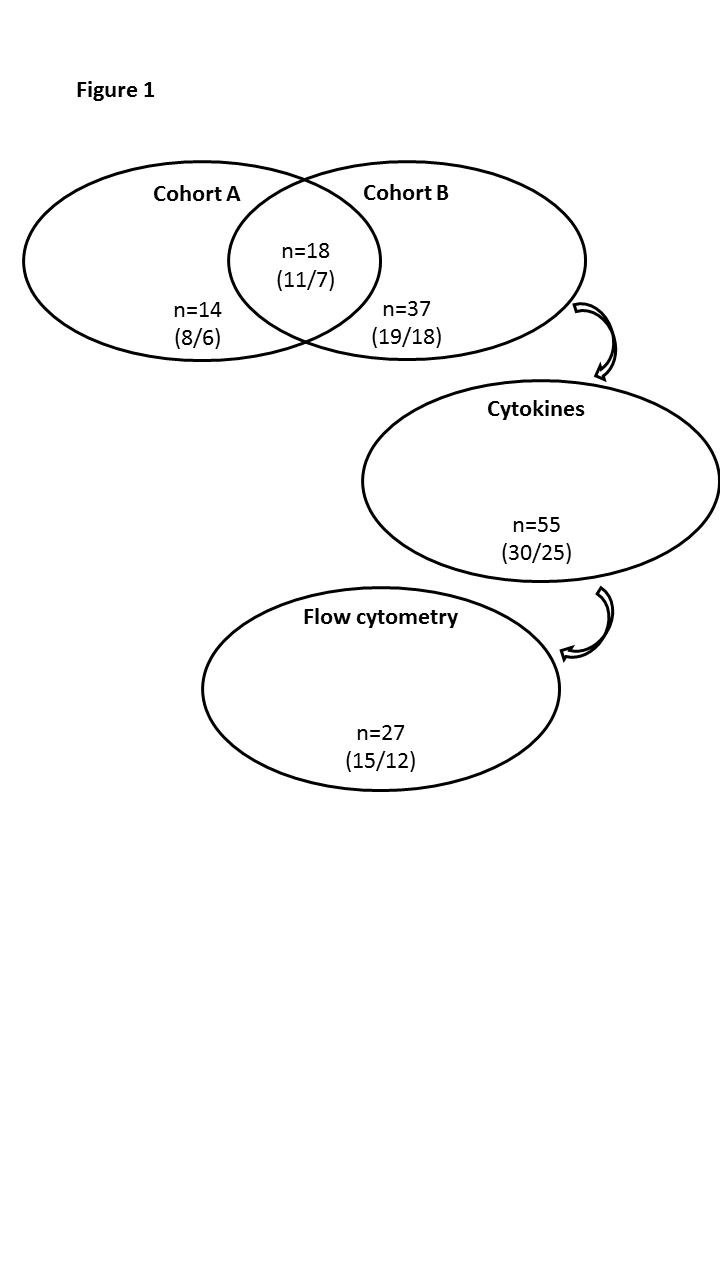
**

**sFigure 1 Study Groups**

Overview of study groups constituting this study.

(Premenopausal women/Postmenopausal women)

**sTable 1** Subject characteristics of study group II (cytokine analyses)

|  | Premenopausal | Postmenopausal |
| --- | --- | --- |
| n | 25 | 30 |
| Age, years  Median (range) | 48 (45 - 54) | 55 (49 - 60) |
| ***Female sex hormones*** | | |
| Estradiol, pmol/l | 170  (98-526) | 60  (51-69) |
| FSH, IU/l | 28.1  (6.7-40.7) | 67.9  (61.4-88.7) |
| ***Body composition*** | | |
| Height, cm | 168  (165-174) | 166  (163-169) |
| Weight, kg | 67.4  (59.6-70.0) | 67.0  (60.8-76.0) |
| BMI | 23.4  (21.6-24.7) | 25.3  (21.1-27.5) |
| Fat mass, kg | 20.0  (15.5-24.6) | 23.9  (18.4-29.1) |
| Visceral fat mass, L | 0.34  (0.21-0.45) | 0.59  (0.33-1.12) |

**sTable 2** Subject characteristics of study group III (flow cytometry analyses)

|  | Premenopausal | Postmenopausal |
| --- | --- | --- |
| n | 15 | 12 |
| Age, years  Median (range) | 47  (45-53) | 55  (49-59) |
| ***Female sex hormones*** | | |
| Estradiol, pmol/l | 158  (63-526) | 64.5  (55.5-81.0) |
| FSH, IU/l | 13.0  (7.1-58.2) | 67.9  (64.9-84.8) |
| ***Body composition*** | | |
| Height, cm | 168  (166-173) | 167  (163-171) |
| Weight, kg | 66.9  (61.1-68.0) | 68.0  (58.4-74.6) |
| BMI | 23.8  (21.2-24.7) | 24.4  (21.6-26.1) |
| Fat mass, kg | 19.9  (15.5-23.2) | 20.4  (17.3-25.8) |
| Visceral fat mass, L | 0.36  (0.17-0.65) | 0.41  (0.31-0.99) |
