# Supplementary material for: Increased systemic inflammation and altered distribution of T-cell subsets in postmenopausal women
Source: PLoS One. 2020 Jun 23;15(6):e0235174. doi: 10.1371/journal.pone.0235174 (PMC7310708; doi:10.1371/journal.pone.0235174)

**S2 APPENDIX: GATING STRATEGIES**

***sTable 3.*** Cell surface markers and gating strategy.

|  | **Cell surface markers and gating strategy** |
| --- | --- |
| Naïve T-cells | Live cells 🡪 single cells 🡪 CD3+ cells 🡪 CD4+/CD8+ cells 🡪 CD45RA+CCR7+ |
| Effector T-cells | Live cells 🡪 single cells 🡪 CD3+ cells 🡪 CD4+/CD8+ cells 🡪 CD45RA+CCR7- |
| Central memory T-cells | Live cells 🡪 single cells 🡪 CD3+ cells 🡪 CD4+/CD8+ cells 🡪 CD45RA-CCR7+ |
| Effector memory T-cells | Live cells 🡪 single cells 🡪 CD3+ cells 🡪 CD4+/CD8+ cells 🡪 CD45RA-CCR7- |
| Activated T-cells | Live cells 🡪 single cells 🡪 CD3+ cells 🡪 CD4+/CD8+ cells 🡪 CD38+HLA-DR+ |
| Senescent T-cells | Live cells 🡪 single cells 🡪 CD3+ cells 🡪 CD4+/CD8+ cells 🡪 CD28-CD57+ |
| Exhausted T-cells | Live cells 🡪 single cells 🡪 CD3+ cells 🡪 CD4+/CD8+ cells 🡪  PD-1+ |

**sFigure 2** Gating strategy from random subject included in the study


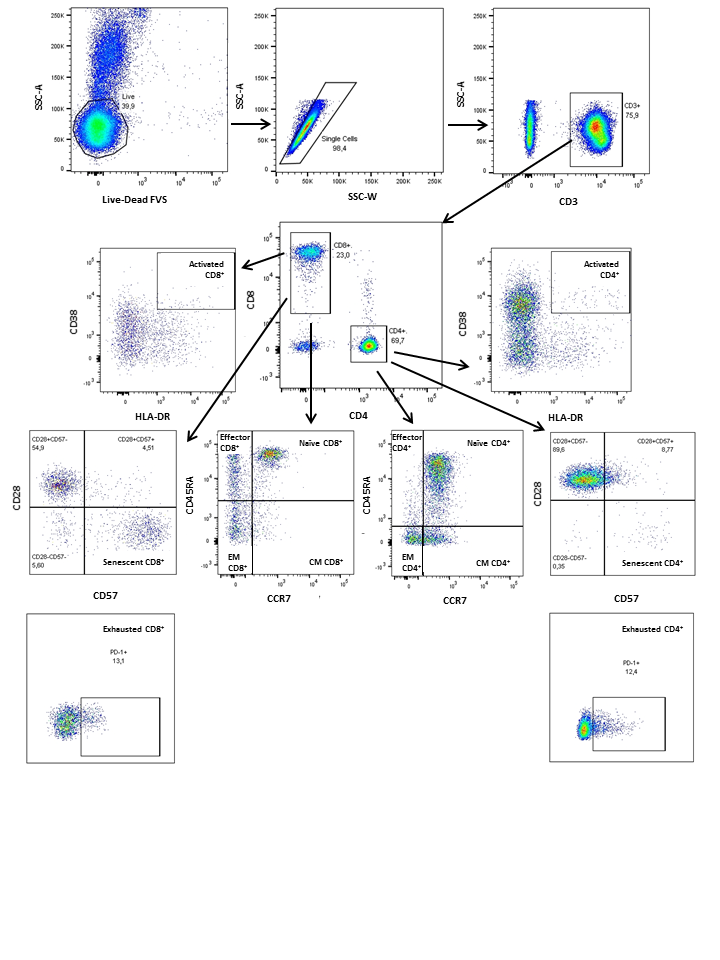

Supplement: S2 Appendix — (DOCX) [file pone.0235174.s002.docx]
